# Supplementary material for: Learning curve and surgical outcome of robotic assisted colorectal surgery with ERAS program
Source: Sci Rep. 2022 Nov 29;12:20566. doi: 10.1038/s41598-022-24665-w (PMC9709162; doi:10.1038/s41598-022-24665-w)
Supplement: Supplementary file 1 — Supplementary Information 1. [file 41598_2022_24665_MOESM1_ESM.zip › LSC Raw data-Ñ[▒K/145/P_ñw╝╨░O▒Kñσ.pdf]

[redacted]

Pathologic diagnosis:
1. Rectum, laparoscopic anterior resection --- Mucinous adenocarcinoma, moderately differentiated, arising in a tubulovillous adenoma. Tumor invades through the visceral peritoneum, and metastasizes to perirectal lymph nodes (5/25). The bilateral cut ends are free of tumor.
2. IMA root, biopsy --- Unremarkable fibroadipose tissue. No lymphoid tissue seen.
3. Colon, cut end, distal, resection --- Negative for malignancy.
4. Peritoneum, biopsy --- Metastatic mucinous adenocarcinoma, moderately differentiated.

Ancillary study for diagnosis:
Re-cut and IHC stains for PMS2 and MSH6 are done on section K. All above markers are preserved.

Prognostic and predictive factor:
1. Tumor size: 6.5x5x5 cm.
2. Depth of invasion: Visceral peritoneum (including tumor continuous with serosal surface through area of inflammation).
3. Histologic grade: G2: Moderately differentiated.
4. Angiolymphatic invasion: Present.
5. Perineural invasion: Not identified.
6. Tumor deposits (discontinuous extramural extension): Present, specify number of deposits: 2.
7. Macroscopic tumor perforation: Not identified.
8. Surgical Margins:
a) Proximal Margin: Uninvolved by invasive carcinoma, distance of tumor from margin: 145 mm.
b) Distal Margin: Uninvolved by invasive carcinoma, distance of tumor from margin: 32 mm.
c) Radial or Mesenteric Margin: Uninvolved by invasive carcinoma, distance of tumor from margin: 3 mm.
9. Lymph node status: Involved (5/25).
10. Extranodal involvement: Present.
11. Tumor regression grading S/P CCRT: No known presurgical therapy.
12. Type of polyp in which invasive carcinoma arose: Tubulovillous adenoma.
13. Additional pathologic findings: Not identified.
14. Pathological TNM Stage: pT4aN2aM1c (According to the eighth edition, American Joint Committee on Cancer Staging Guidelines for Tumors).
15. TNM descriptors: Not applicable.

Gross description:
The specimen consists of 1) a segment of rectum, 23 cm in length and 4 cm in proximal cut end circumference, 6 cm in distal circumference, and perirectal soft tissue. A gray tan firm annular elevated and centrally ulcerative lesion, 6.5x5.0x5.0 cm, is noted, and it is , 14.5 cm and 3.2 cm respectively to the proximal and distal cut ends. On cross section, the lesion involves the underlying perirectal soft tissue with grossly 3 mm to the radial margin. The lesion is less than 1 mm to serosa, and serosal retraction is also noted. The both cut ends are free of the lesion grossly. The serosa is inked by blue dye, and the radial margin is inked by green dye. 2) IMA root, a piece of tan soft tissue, 5.0x1.5x1.3 cm. 3) Distal cut end, a piece of tan mucosa-covered soft tissue, 1.6x1.6x0.7 cm. 4) "Peritoneum", a piece of tan soft tissue, 1.2x0.8x0.5 cm. Representative part for section: A) proximal cut end B-C) distal cut end D-Q) lesion (mapping: D-J and K-Q) R) lesion with colon mucosa S-T) specimen 2 W-Y) lymph nodes; All for section: U) specimen 3 V) specimen 4.

Note:
1. The interpretation of MMR panel and recommended studies are as below: (1) No loss of nuclear expression of MMR proteins: low probability of microsatellite instability-high (MSI-H) . (2) Loss of nuclear expression of MLH1 and PMS2: testing for methylation of the MLH1 promoter and/or mutation of BRAF is indicated (the presence of a BRAF V600E mutation and/or MLH1 methylation suggests that the tumor

BRAF V600E mutation and/or MLH1 methylation suggests that the tumor
is sporadic and germline evaluation is probably not indicated;
absence of both MLH1 methylation and of BRAF V600E mutation suggests
the possibility of Lynch syndrome, and sequencing and/or large
deletion/duplication testing of germline MLH1 may be indicated). (3)
Loss of nuclear expression of MSH2 and MSH6: high probability of
Lynch syndrome (sequencing and/or large deletion/duplication testing
of germline MSH2 may be indicated, and, if negative, sequencing
and/or large deletion/duplication testing of germline MSH6 may be
indicated). (4) Loss of nuclear expression of MSH6 only: high
probability of Lynch syndrome (sequencing and/or large
deletion/duplication testing of germline MSH6 may be indicated). (5)
Loss of nuclear expression of PMS2 only: high probability of Lynch
syndrome (sequencing and/or large deletion/duplication testing of
germline PMS2 may be indicated). (6) Please note: There are
exceptions to the above IHC interpretations. These results should not
be considered in isolation, and clinical correlation with genetic
counseling is recommended to assess the need for germline testing.
2. This case has been peer reviewed by two doctors.
#T-59600_2 #M-84803_2 2 1105 S0000F
#T-C4560_2 #M-81406_2 2 1105 000000

已電子簽章

敬告：本單僅供醫師參考使用，不具任何申請效力！！
請病患妥為保管，若不慎遺失需自負個人資料外洩責任！
